# Is Musculoskeletal Ultrasonography an Operator-Dependent Method or a Fast and Reliably Teachable Diagnostic Tool? Interreader Agreements of Three Ultrasonographers with Different Training Levels

**DOI:** 10.1155/2010/164518

**Published:** 2010-12-09

**Authors:** Sarah Ohrndorf, Lydia Naumann, Jessica Grundey, Tanja Scheel, Alexander K. Scheel, Carola Werner, Marina Backhaus

**Affiliations:** ^1^Department of Rheumatology and Clinical Immunology, Charité-University Medicine Berlin, Charitéplatz 1, 10117 Berlin, Germany; ^2^Department of Clinical Neurophysiology, Georg-August-University Goettingen, Robert Koch Street 40, 37075 Goettingen, Germany; ^3^Department of Rheumatology, Johann-Wolfgang-Goethe University Frankfurt, Theodor-Stern-Kai 7, 60590 Frankfurt, Germany; ^4^HELIOS Research Center, Institute of Medical Statistics, 10117 Berlin, Germany

## Abstract

*Objectives*. To assess interreader agreements and a learning curve between three (senior, junior, and beginner) different experienced musculoskeletal ultrasonographers. Senior served as the imaging “gold standard”. *Methods*. Clinically dominant joints (finger, shoulder, knee, tibiotalar, and talonavicular) of 15 rheumatoid arthritis (RA) patients were examined by three different experienced ultrasonographers (senior 10 years, junior 10 months, and beginner one month). Each patient's ultrasonographic findings were reported unaware of the other investigators' results. *κ* coefficients, percentage agreements, sensitivities, and specificities were calculated. *Results*. 120 joints of 15 RA patients were evaluated. Comparing junior's and beginner's results each to the senior's findings, the overall *κ* for all examined joints was 0.83 (93%) for junior and 0.43 (76%) for beginner. Regarding the different joints, junior's findings correlate very well with the senior's findings (finger joints: *κ* = 0.82; shoulder: *κ* = 0.9; knee: *κ* = 0.74; tibiotalar joint: *κ* = 0.84; talonavicular joint: *κ* = 0.84) while beginner's findings just showed fair to moderate agreements (finger joints: *κ* = 0.4; shoulder: *κ* = 0.42; knee: *κ* = 0.4; tibiotalar joint: *κ* = 0.59; talonavicular joint: *κ* = 0.35). In total, beginner's results clearly improved from *κ* = 0.34 (agreement of 67%) at baseline to *κ* = 0.78 (agreement of 89%) at the end of the evaluation period. *Conclusions*. Ultrasonographic evaluation of a ten-month-experienced investigator in comparison to a senior ultrasonographer was of substantial agreement. Agreements between a beginner and a highly experienced ultrasonographer were only fair at the beginning, but during the study including ultrasonographical sessions of 15 RA patients, the beginner clearly improved in musculoskeletal ultrasonography.

## 1. Introduction

Rheumatoid arthritis (RA) is characterised by synovitis and erosions of small finger joints, though large joints are also commonly affected. Due to technical improvements, musculoskeletal ultrasonography (US) has become an established method to detect soft tissue inflammatory process and early superficial bone lesions in patients with RA. In comparison to other imaging methods as diagnostic tools in rheumatology, US has remarkable advantages like easy and quick access, noninvasiveness, inexpensiveness, ability to scan multiple joints, repeatability and high patient acceptability [1]. However, it has been stated but not sufficiently investigated that musculoskeletal US is one of the most operator-dependent imaging techniques [2, 3]. The inter- and intraobserver variations have only been tested in a minority of studies [2–4]. In recent studies of the European League against Rheumatism (EULAR) working group for imaging in RA, interobserver reliabilities, sensitivities, and specificities in comparison with MRI were found to be moderate to good [2, 3]. Nevertheless, further standardisation of US scanning techniques and definitions of different pathological lesions are needed to increase the interobserver agreement in musculoskeletal US so that results of US reports can be compared in multicenter studies. 

Further studies are also necessary to explore the time needed to assess practical US skills in order to perform a sufficient musculoskeletal US scan. A learning curve has been assessed by D'Agostino et al. who evaluated synovitis in MCP, PIP, and MTP joints. They found that at least 70 examinations were necessary to develop ultrasonographical competence in detecting synovitis in small joints [5]. Bone erosions and other commonly affected joints were not included in this study. Hence, we performed a study with three (senior, junior, and beginner) different experienced ultrasonographers evaluating small finger, tibiotalar, and talonavicular joints as well as large joints like the shoulder and knee of RA patients in order to differentiate between the learning ability with regard to the different joints and joint pathologies. Junior's and beginner's results were compared to the senior's results who served as the imaging “gold standard”. 

## 2. Patients and Methods

### 2.1. Patients and Joint Regions

Fifteen patients with RA (ten female, five male, mean (SD) age 58 (±14,9) years, range 29 to 84) according to the American College of Rheumatology criteria [6] were recruited from the Rheumatology Outpatient Clinic of the University Hospital Goettingen, Germany. They were examined once by three different experienced ultrasonographers each on the same examination day during an evaluation time of two months. Altogether, 120 of clinically dominant joints have been assessed in this study. These were finger joints (MCP II and III PIP II and III), shoulder, knee, tibiotalar, and talonavicular joints.

### 2.2. Musculoskeletal Ultrasonographers

US was performed by three different experienced ultrasonographers: the *senior* ultrasonographer (AKS) was working as an MD in musculoskeletal US for ten years and as a member of the EULAR and OMERACT US expert group; he was therefore considered as a US specialist. The *junior *ultrasonographer (JG) also worked as an MD at this study's duration with ten months of experience in the field of musculoskeletal US. She was using US as a routine diagnostic tool as well as in doing clinical research studies, especially in the scanning of finger and toe joints. In average, she scanned two to three patients per day. The *beginner *ultrasonographer (SO) was still a medical student during this study with one month of musculoskeletal US experience. She underwent one month (12 hours per week) of practical ultrasonographical training sessions (“hands-on” training and didactic instructions of standard scans) before the beginning of the study and had therefore done 50 hours of US training before this study's onset. 

The three ultrasonographers reported and documented their US findings independently and unaware of the other investigators' results at the same visit of each patient.

### 2.3. Ultrasonographic Investigation

US was performed with an Esaote Technos MPX machine (Esaote S.p.A., Genova, Italy) with two different linear array transducers (8–14 MHz for finger, tibiotalar, and talonavicular joints and 4–13 MHz for shoulder and knee joints). Each ultrasonographer evaluated clinically dominant finger, shoulder, knee, tibiotalar, and talonavicular joints according to the German [7–10] and EULAR [1] standard scans by using grey-scale (GS) US.

**Figure 1:**
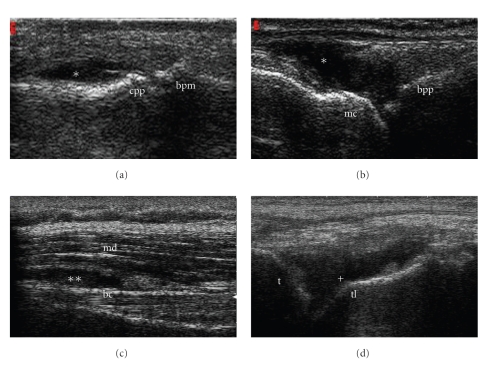
Longitudinal grey-scale ultrasound images of the third proximal interphalangeal joint from dorsal view with synovitis* (a), the third metacarpophalangeal joint from dorsal view with synovitis* (b), the shoulder joint from anterior view with subdeltoid bursitis**, and the tibiotalar joint with effusion^+^ (d). Abbreviations; cpp: Caput of phalanx proximalis; bpm: Basis of phalanx medialis; bpp: Basis of phalanx proximalis; mc: Caput of metacarpus; md: Deltoid muscle; bc: Tendon of long biceps muscle; tl: Talus; t: Tibia.

The finger joints MCP II, III and PIP II, III were examined both for synovitis (Figures 1(a) and 1(b)) and for erosions, each from the dorsal and from the palmar view. The MCP joint II was also scanned laterally from radial in terms of erosions. Synovitis was defined as both synovial hypertrophy and effusion [11]. An interruption of the bone surface in two perpendicular planes was described as an erosion as defined by the OMERACT group [12]. 

For the shoulder joint, emphasis was taken on the following pathologies. Firstly, tenosynovitis of the long biceps tendon was described if there was a hypo-/anechoic thickened tissue with or without fluid within the tendon sheath, which is seen in two perpendicular planes [12]. Secondly, we looked for subdeltoid bursitis in the anterior (Figure 1(c)), lateral, and dorsal view. Further, the joint was evaluated for partial/full rotator cuff rupture. The humeral head surface was also evaluated for erosions according to the OMERACT definition for erosion [12]. A pathologic distension of the joint capsule with an intraarticular effusion and/or synovial proliferation was defined as synovitis in the dorsal and anterior region.

The evaluated pathologies for the knee joint were suprapatellar effusion, synovitis, and erosions of the medial and lateral joint recess and popliteal cysts according to German standard scans for knee joint examination [9]. 

The tibiotalar joint was examined both for effusion (Figure 1(d)) and for erosion, while the talonavicular joint was just assessed for effusion after definition of US ankle and foot examination [10]. The pathologies for the ultrasonographic investigation are listed in Table 1. All of the assessed pathologies have been evaluated on a qualitative yes/no (1/0) basis.

### 2.4. Interreader and Learning Curve Sessions

Three different experienced ultrasonographers evaluated 15 RA patients during an examination time of two months. On the one hand, interreader results were evaluated with regard to the different joints and joint pathologies. On the other hand, an overall *κ* and agreement for the findings of each of the 15 US examination sessions were calculated. In case of the beginner's results, a learning curve was developed.

### 2.5. Statistical Analysis

The junior's and the beginner's results were compared with the senior's results using *κ* coefficients, level of agreement in a percentage (%), sensitivities, and specificities calculated by the statistical software package SAS 8.02 (SAS Institute Inc., Cary, NC, USA). The *κ* coefficients were divided as follows: *κ* < 0.0: poor, *κ* = 0–0.20: slight, *κ* = 0.21–0.40: fair, *κ* = 0.41–0.60: moderate, *κ* = 0.61–0.80: substantial, and *κ* = 0.81–1.0: almost perfect agreement [13]. The interreader agreements refer to the learning time period as well as to the differently examined joint regions.

## 3. Results

Comparing junior's and beginner's results each in comparison to the senior's findings, the overall *κ* (overall percentage agreement) for all examined joints was 0.83 (93%) for junior and 0.43 (76%) for beginner. 

Regarding the different joints, calculations showed substantial to almost perfect agreements for the *junior* investigator: finger joints: *κ* = 0.82, sensitivity 95%, specificity 89%; shoulder: *κ* = 0.9, sensitivity 90%, specificity 98%; knee: *κ* = 0.74, sensitivity 91%, specificity 93%; tibiotalar joint*: κ* = 0.84, sensitivity 100%, specificity 90%; talonavicular joint: *κ* = 0.84, sensitivity 100%, specificity 80%. 

Beginner investigator's findings showed moderate to fair agreements: finger joints: *κ* = 0.40, sensitivity 69%, specificity 74%; shoulder: *κ* = 0.42, sensitivity 58%, specificity 95%; knee: *κ* = 0.40, sensitivity 60%, specificity 93%; tibiotalar joint: *κ* = 0.59, sensitivity 76%, specificity 85%; talonavicular joint: *κ* = 0.35, sensitivity 60%, specificity 80%.

Results of agreements concerning the examined pathologies in each joint region are presented in Tables 2(a) and 2(b). Junior reached perfect *κ* values in detecting rotator cuff rupture and popliteal cyst (each *κ* = 1) whereas the beginner's agreements resulted in just a *κ*-value of 0.3 for rotator cuff rupture and, respectively, *κ* = 0.76 for the diagnosis of popliteal cyst.

**Figure 2:**
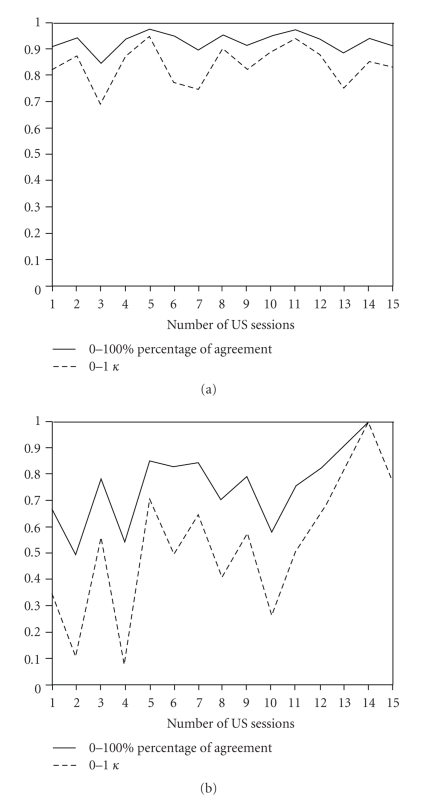
(a) Interreader agreements between senior and junior. Grade of agreement and *κ* coefficients from the first to the 15th US session for the junior investigator. (b) Intereader agreements between senior and beginner (learning curve). Grade of agreement and *κ* coefficients from the first to the 15th US session for the beginner investigator.

The interreader agreements between junior and senior according to each US evaluation session (all in all 15) were substantial to almost perfect (mean *κ* = 0.83; mean agreement = 93%). During the study, the junior ultrasonographer could constantly keep high agreement levels (Figure 2(a)). The interreader agreements between beginner and senior clearly improved from *κ* = 0.34 (agreement 67%) at first date of the US evaluation session to *κ* = 0.78 (agreement 89%) at the end of this 2 months evaluation period presented in a learning curve (Figure 2(b)). US improvement of the beginner especially is presented after the 10th evaluation date. 

## 4. Discussion

During the last decade, musculoskeletal US has become an indispensable diagnostic tool in the management of rheumatic diseases. In patients with RA especially it is important for both diagnosis and disease monitoring. It is widely used as an important outcome measure in therapeutic trials in RA [14–17]. Main criticism of US is that it is one of the most operator-dependent imaging methods. However, Scheel et al. were recently able to show moderate to good interreader agreements in the first interobserver variability study performed by 14 experts of the EULAR working group [2]. Confirming these findings in a larger study, Naredo et al. also found moderate to good interobserver reliabilities between 23 European musculoskeletal ultrasound experts [3]. Nevertheless, further standardisation of US scanning techniques and definitions of different pathological lesions are needed to increase the interobserver agreement in musculoskeletal US [18–20]. D'Agostino et al. performed the first study examining the rate at which rheumatologists with little or no experience in musculoskeletal US develop adequate skills to undertake a US evaluation. They showed that 70 examinations are necessary (including training sessions) to assess synovitis of the small MCP, PIP, and MTP joints accurately [5].

In our study, three different experienced ultrasonographers evaluated small *and* large joints which are commonly affected in RA. We decided to assess clinically dominant finger joints, shoulder, knee, tibiotalar, and talonavicular joints for synovitis, erosions, and other typical RA joint pathologies. The wrist was excluded because erosions are difficult to detect and to distinguish from physiological irregularities. A ten-month-experienced musculoskeletal ultrasonographer compared to a ten-year-experienced investigator reached substantial to almost perfect agreements (mean *κ* = 0.83). In the detection of rotator cuff ruptures and popliteal cysts, the junior even reached a *κ*-value of 1. In contrast to our earlier study [2] though, in which the knee showed a *κ*-value of 1, the ten-month-experienced ultrasonographer this time just received an agreement of *κ* = 0.74 for the knee. As Table 2(a) presents, this was because of the fact that the junior ultrasonographer reached only *κ* = 0.57 in detecting suprapatellar effusion and *κ* = 0.63 in detecting erosions in the knee joint. This might especially be explained due to difficulties in the detection of small fluid in the suprapatellar recess (maybe loss of dynamic examination by no contraction of quadriceps muscle). In addition, the junior ultrasonographer mainly has had experience in assessing small finger and toe joints by doing research studies in this field. Consequently, the junior got substantial to almost perfect agreements in the finger joint examination of erosions (*κ* range 0.71–1.0) and synovitis (*κ* range 0.62–1.0), except MCP III. In the examination of MCP joint III, *κ*-values of 0.48 (dorsal) and 0.62 (palmar) in detecting synovitis were just reached by the junior (Table 2(a)). Interestingly, Szkudlarek et al. also found the highest US intervariability in the MCP joint III (ICC = 0.57) in comparison to other small joints (MCP II, PIP II, MTP I, II) [4]. In contrast to the very good results reached by the junior investigator in the examination of erosions, the beginner's results were partly extremely poor, especially in the detection of erosions in the radial MCP II joint part (Table 2(b)). This is a region of high interest for the detection of early erosions in RA. Consequently, accurate assessment through special training of this region is strongly needed to improve the detection of erosions. In regard to the included joints, the most difficult joint to assess seemed to be the talonavicular joint with a fair kappa agreement of 0.34. This can certainly be explained by the fact that small amounts of fluid were not detected by the beginner, especially at the beginning of the study. However, during the study period which included ultrasonography of 15 patients with RA and the examination of 120 RA joints, the beginner's ultrasonographical competence clearly improved, and the beginner gained substantial agreement with the senior (from *κ* = 0.34 to *κ* = 0.78).

In this study, junior and beginner ultrasonographers have both been taught by the same senior, who served as the imaging “gold standard”, a constellation that implicates the risk of a possible less objectivity of this study. Furthermore, an intrareader examination was not provided in this study. Larger studies with students of different US training levels from various US backgrounds are needed to confirm our results. Another critical point might include the fact that the reliability of power Doppler US as an emerging important tool in the assessment of synovitis activity was not proven in this study. 

Taking our study results together, we were able to show that a US investigator with ten months of experience reached good to almost perfect agreement with a ten-year-experienced senior ultrasonographer and that a little experienced ultrasonographer substantially improved US competence within a period of two months suggesting that a relatively short teaching time can already lead to sufficient diagnostic US findings in grey-scale musculoskeletal ultrasonography. Therefore, the main criticism against musculoskeletal US as an operator-dependent and difficult to learn method might be attenuated.

## Figures and Tables

**Table 1:** Ultrasonographic investigation.

Anatomical structure	Ultrasonographic findings
*Finger joints*	
MCP II+III (dorsal+palmar+ radial in MCP II)	Synovitis (effusion and synovial proliferation) and erosions
PIP II+III (dorsal+palmar)	

*Shoulder*	
Glenohumeral joint (anterior and posterior recess)	Synovitis
Humeral head (anterior and posterior aspect)	Erosions
Long biceps tendon	Tenosynovitis
Subscapularis, supraspinatus, infraspinatus tendon	partly/full rotator cuff rupture
Subdeltoid bursae	Bursitis

*Knee*	
Suprapatellar recess	Effusion
Bone surface (medial + lateral)	Erosions
Gastrocnemius-Semimembranosus bursa	Popliteal cyst

*Tibiotalar joint*	Synovitis and erosions
*Talonavicular joint*	Synovitis

**Table:** (a) Results for the junior investigator; na = not available.

Joints + pathologies	Specificity	Sensitivity	*κ*	Agreement (%)	Senior's results 0/1
*Finger joints *(synovitis)*: *					
MCP II dorsal	100%	100%	1.0	100%	5/10
MCP II palmar	75%	100%	0.81	93.3%	4/11
MCP III dorsal	50%	100%	0.48	73.3%	8/7
MCP III palmar	66.7%	100%	0.62	80%	9/6
PIP II dorsal	92.3%	100%	0.76	93.3%	13/2
PIP II palmar	100%	88.9%	0.86	93.3%	6/9
PIP III dorsal	100%	71.4%	0.73	86.7%	8/7
PIP III palmar	100%	87.5%	0.87	93.3%	7/8
MCP II radial	100%	100%	1.0	100%	1/10; na = 4

*Finger joints *(erosion):					
MCP II dorsal	83.3%	100%	0.86	93.3%	6/9
MCP II palmar	75%	100%	0.82	93.3%	4/11
MCP III dorsal	85.7%	87.5%	0.73	86.7%	7/8
MCP III palmar	83.3%	100%	0.86	93.3%	6/9
PIP II dorsal	100%	90.9%	0.84	93.3%	4/11
PIP II palmar	66.7%	100%	0.71	86.7%	6/9
PIP III dorsal	100%	91.7%	0.81	93.3%	3/12
PIP III palmar	100%	100%	1.0	100%	6/9
MCP II radial	100%	100%	1.0	100%	1/10; na = 4

*Shoulder*					
Anterior synovitis	100%	66.7%	0.76	93.3%	12/3
Posterior synovitis	100%	100%	1.0	100%	13/2
Anterior erosion	100%	100%	1.0	100%	1/14
Posterior erosion	100%	100%	1.0	100%	1/14
Tenosynovitis of biceps tendon	88.9%	83.3%	0.72	86.7%	9/6
Rotator cuff rupture	100%	100%	1.0	100%	7/4
Subdeltoid bursitis	100%	80%	0.84	93.3%	10/5

*Knee*					
Suprapatellar effusion	80%	80%	0.57	80%	5/10
Erosion (medial + lateral)	100%	92.9%	0.63	93.3%	1/14
Popliteal cyst	100%	100%	1.0	100%	12/3

*Tibiotalar joint*					
Synovitis	91.7%	100%	0.81	93.3%	12/3
Erosion	87.5%	100%	0.87	93.3%	8/7
*Talonavicular joint*					
Synovitis	80%	100%	0.84	93.3%	5/10

**Table:** (b) Results for the beginner investigator; na = not available

Joints + pathologies	Specificity	Sensitivity	*κ*	Agreement (%)	Senior's results 0/1
*Finger joints (synovitis):*					
MCP II dorsal	100%	100%	1.0	100%	5/10
MCP II palmar	75%	81.8%	0.53	80%	4/11
MCP III dorsal	75%	85.7%	0.60	80%	8/7
MCP III palmar	88.9%	66.7%	0.57	80%	9/6
PIP II dorsal	84.6%	0%	−0.15	73.3%	13/2
PIP II palmar	83.3%	66.7%	0.47	73.3%	6/9
PIP III dorsal	87.5%	42.9%	0.31	66.7%	8/7
PIP III palmar	85.7%	37.5%	0.22	60%	7/8
MCP II radial	100%	100%	1.0	100%	1/10; na = 4

*Finger joints (erosion):*					
MCP II dorsal	83.3%	55.6%	0.36	66.7%	6/9
MCP II palmar	75%	54.6%	0.22	60%	4/11
MCP III dorsal	71.4%	37.5%	0.09	53.3%	7/8
MCP III palmar	100%	88.9%	0.86	93.3%	6/9
PIP II dorsal	50%	72.7%	0.21	66.7%	4/11
PIP II palmar	33.3%	77.8%	0.12	60%	6/9
PIP III dorsal	100%	75%	0.55	80%	3/12
PIP III palmar	33.3%	100%	0.38	73.3%	6/9
MCP II radial	0%	90%	−0.1	81.8%	1/10; na = 4

*Shoulder*					
Anterior synovitis	100%	66.7%	0.76	93.3%	12/3
Posterior synovitis	84.6%	50%	0.29	80%	13/2
Anterior erosion	100%	78.6%	0.33	80%	1/14
Posterior erosion	100%	78.6%	0.33	80%	1/14
Tenosynovitis of biceps tendon	77.8%	50%	0.29	66.7%	9/6
Rotator cuff rupture	100%	25%	0.3	72.7%	7/4
Subdeltoid bursitis	100%	60%	0.67	86.7%	10/5

*Knee*					
Suprapatellar effusion	80%	50%	0.25	60%	5/10
Erosion (medial + lateral)	100%	64.3%	0.19	66.7%	1/14
Popliteal cyst	100%	66.7%	0.76	93.3%	12/3

*Tibiotalar joint*					
Synovitis	83.3%	66.7%	0.44	80%	12/3
Erosion	87.5%	85.7%	0.73	86.7%	8/7

*Talonavicular joint*					
Synovitis	80%	60%	0.35	66.7%	5/10

## References

[1] Backhaus M, Burmester G-R, Gerber T (2001). Guidelines for musculoskeletal ultrasound in rheumatology. *Annals of the Rheumatic Diseases*.

[2] Scheel AK, Schmidt WA, Hermann K-GA (2005). Interobserver reliability of rheumatologists performing musculoskeletal ultrasonography: results from a EULAR "Train the trainers" course. *Annals of the Rheumatic Diseases*.

[3] Naredo E, Möller I, Moragues C (2006). Interobserver reliability in musculoskeletal ultrasonography: results from a “Teach the Teachers” rheumatologist course. *Annals of the Rheumatic Diseases*.

[4] Szkudlarek M, Court-Payen M, Jacobsen S, Klarlund M, Thomsen HS, Østergaard M (2003). Interobserver agreement in ultrasonography of the finger and toe joints in rheumatoid arthritis. *Arthritis and Rheumatism*.

[5] D’Agostino M-A, Maillefert J-F, Said-Nahal R, Breban M, Ravaud P, Dougados M (2004). Detection of small joint synovitis by ultrasonography: the learning curve of rheumatologists. *Annals of the Rheumatic Diseases*.

[6] Arnett FC, Edworthy SM, Bloch DA (1988). The American Rheumatism Association 1987 revised criteria for the classification of rheumatoid arthritis. *Arthritis and Rheumatism*.

[7] Backhaus M, Schmidt WA, Mellerowicz H (2002). Technique and diagnostic value of musculoskeletal ultrasonography in rheumatology—part 6: ultrasonography of the wrist/hand. *Zeitschrift fur Rheumatologie*.

[8] Mellerowicz H, Hauer RW, Schmidt WA (2002). Technique and diagnostic value of musculoskeletal ultrasonography in rheumatology—part 5: ultrasonography of the shoulder. *Zeitschrift fur Rheumatologie*.

[9] Hauer RW, Schmidt WA, Bohl-Bühler M (2001). Technique and value of arthrosonography in rheumatologic diagnosis. 1: ultrasound diagnosis of the knee joint. *Zeitschrift für Rheumatologie*.

[10] Schmidt WA, Hauer RW, Banzer D (2002). Technique and value of arthrosonography in rheumatologic diagnosis—3: ultrasound diagnosis of the ankle joint, foot and toes. *Zeitschrift für Rheumatologie*.

[11] Scheel AK, Hermann K-GA, Kahler E (2005). A novel ultrasonographic synovitis scoring system suitable for analyzing finger joint inflammation in rheumatoid arthritis. *Arthritis and Rheumatism*.

[12] Wakefield RJ, Balint P, Szkudlarek M (2005). Proceedings from the OMERACT Special Interest Group for Musculoskeletal Ultrasound including definitions for ultrasonographic pathology. *Journal of Rheumatology*.

[13] Landis JR, Koch GG (1977). The measurement of observer agreement for categorical data. *Biometrics*.

[14] Backhaus M, Burmester GR, Sandrock D (2002). Prospective two year follow up study comparing novel and conventional imaging procedures in patients with arthritic finger joints. *Annals of the Rheumatic Diseases*.

[15] Hermann K-GA, Backhaus M, Schneider U (2003). Rheumatoid arthritis of the shoulder joint: comparison of conventional radiography, ultrasound, and dynamic contrast-enhanced magnetic resonance imaging. *Arthritis and Rheumatism*.

[16] Szkudlarek M, Narvestad E, Klarlund M, Court-Payen M, Thomsen HS, Østergaard M (2004). Ultrasonography of the metatarsophalangeal joints in rheumatoid arthritis: comparison with magnetic resonance imaging, conventional radiography, and clinical examination. *Arthritis and Rheumatism*.

[17] Scheel AK, Hermann K-GA, Ohrndorf S (2006). Prospective 7 year follow up imaging study comparing radiography, ultrasonography, and magnetic resonance imaging in rheumatoid arthritis finger joints. *Annals of the Rheumatic Diseases*.

[18] Østergaard M, Wiell C (2004). Ultrasonography in rheumatoid arthritis: a very promising method still needing more validation. *Current Opinion in Rheumatology*.

[19] Brown AK, O’Connor PJ, Wakefield RJ, Roberts TE, Karim Z, Emery P (2004). Practice, training, and assessment among experts performing musculoskeletal ultrasonography: toward the development of an international consensus of educational standards for ultrasonography for rheumatologists. *Arthritis Care and Research*.

[20] Schmidt WA, Schmidt H, Schicke B, Gromnica-Ihle E (2004). Standard reference values for musculoskeletal ultrasonography. *Annals of the Rheumatic Diseases*.

